# An assessment of human impacts on endangered red pandas (*Ailurus fulgens*) living in the Himalaya

**DOI:** 10.1002/ece3.5797

**Published:** 2019-11-07

**Authors:** Saroj Panthi, Tiejun Wang, Yiwen Sun, Arjun Thapa

**Affiliations:** ^1^ Ministry of Industry, Tourism Forest, and Environment Pokhara Nepal; ^2^ Department of Natural Resources Faculty of Geo‐Information Science and Earth Observation (ITC) University of Twente Enschede The Netherlands; ^3^ Small Mammals Conservation and Research Foundation Kathmandu Nepal

**Keywords:** anthropogenic variables, distance to path, ecological niche model, habitat suitability, human population density, livestock density

## Abstract

Anthropogenic factors play an important role in shaping the distribution of wildlife species and their habitats, and understanding the influence of human activities on endangered species can be key to improving conservation efforts as well as the implementation of national strategies for sustainable development. Here, we used species distribution modeling to assess human impacts on the endangered red panda (*Ailurus fulgens*) in high‐altitude regions of Nepal. We found that the distance to paths (tracks used by people and animals), livestock density, human population density, and annual mean temperature were the most important factors determining the habitat suitability for red pandas in Nepal. This is the first study that attempts to use comprehensive environmental and anthropogenic variables to predict habitat suitability for the red pandas at a national level. The suitable habitat identified by this study is important and could serve as a baseline for the development of conservation strategies for the red panda in Nepal.

## Graphical abstract

The distance to paths, livestock density, human population density, and annual mean temperature were the most important factors determining the habitat suitability for red pandas in Nepal.

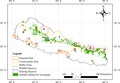

## 1 INTRODUCTION

Global biodiversity has been declining over the last several decades, mainly due to increasing anthropogenic interference (Tittensor et al., 2014). Overexploitation of natural resources and agricultural activities such as crop and livestock production have been identified as major causes of global biodiversity loss (Maxwell, Fuller, Brooks, & Watson, 2016). Habitat loss and degradation represent some of the most significant threats to wildlife species and are closely linked to the expansion of roads and human settlements. Unfortunately, large‐scale effects of these anthropogenic activities remain overlooked. Moreover, human populations are heavily localized at low elevations, with low density at high elevations (Cohen & Small, 1998), and it is generally believed that biodiversity in high‐altitude regions is less disturbed by human activities than those living in low‐altitude regions (Kumar & Ram, 2005; Zhang, Huang, Wang, Liu, & Du, 2016). However, such assertions have not been tested in Nepal, where more than 1,200 human settlements are situated above 3,000 m (Chidi, 2009).

Nepal is remarkable for its rich biodiversity, which is due in part to the country's large variation in elevation (67–8,848 m; MFSC, 2002). The high‐altitude regions in Nepal are not only important for wildlife, but are also essential for the livelihood of local people, allowing for activities such as livestock grazing and collection of nontimber forest products, as well as income from tourism revenue (Aryal, Maraseni, & Cockfield, 2014; Chidi, 2009; DNPWC, 2016; Musa, Hall, & Higham, 2004; Uprety, Poudel, Gurung, Chettri, & Chaudhary, 2017). Livestock grazing in Nepal also increases at higher elevation (Thapa, All, & Yadav, 2016). Previous studies have shown that tourism activities and livestock grazing pose a serious threat to the wildlife and its habitat in this region (Nepal & Nepal, 2004; Sharma, Belant, & Swenson, 2014; Shrestha & Wegge, 2008; Thapa et al., 2016).

The red panda (*Ailurus fulgens*) is a typical high‐altitude animal, living at elevations between 2,200 and 4,800 m (Roberts & Gittleman, 1984). This species is found in the mountains of the Himalayas from western Nepal through northeastern India and Bhutan and into China, Laos and northern Myanmar (Glatston, Wei, Zaw, & Sherpa, 2015). The conservation status of the red panda is “Endangered” on International Union for Conservation of Nature (IUCN) red list (Glatston et al., 2015) and it is included in Appendix 1 of the Convention on International Trade in Endangered Species of Wild Fauna and Flora (CITES) (CITES, 2017). Red pandas are generally shy and solitary animals; they prefer steeper slopes with a high density of fallen logs, shrubs, and bamboo culms (Wei, Feng, Wang, & Hu, 2000), sparse forest (Qi, Hu, Gu, Li, & Wei, 2009) and understory bamboo (Chakraborty et al., 2015; Dorji, Vernes, & Rajaratnam, 2011; Panthi, Aryal, Raubenheimer, Lord, & Adhikari, 2012; Pradhan, Saha, & Khan, 2001; Roberts & Gittleman, 1984). Bamboo leaves and shoots are a major food source (Fei et al., 2017; Hu et al., 2017; Panthi et al., 2012; Panthi, Coogan, Aryal, & Raubenheimer, 2015; Sharma, Swenson, & Belant, 2014; Thapa & Basnet, 2015; Wei, Feng, Wang, Zhou, & Hu, 1999). Although the red panda is protected by international conventions (CITES, 2017) and national law in Nepal (GoN, 1973), its population has continued to decline over the past 30 years (Glatston et al., 2015). The anthropogenic impact on red panda habitat has been identified as a major threat to the conservation of this species in its current distribution range (Acharya et al., 2018; Dendup, Cheng, Lham, & Tenzin, 2017; Dorji, Rajaratnam, & Vernes, 2012; Panthi, Khanal, Acharya, Aryal, & Srivathsa, 2017). A large number of cattle, herders, and their guard dogs have also been responsible for disturbance to red pandas and their habitats (Yonzon & Hunter, 1991a).

To protect red panda habitat, managers need broad‐scale geographic information. While numerous studies have been conducted to assess habitats, conservation threats, and diets of red pandas at local scales in Nepal (Bista et al., 2017; Bista, Panthi, & Weiskopf, 2018; Panthi et al., 2012, 2015, 2017; Sharma, Swenson, et al., 2014; Thapa & Basnet, 2015), few studies have investigated the species distribution and threats to their habitat at national and regional scales (Acharya et al., 2018; Kandel et al., 2015; Thapa et al., 2018). Anthropogenic factors play an important role in shaping the distribution of wildlife species and their habitats (Lewis et al., 2017), and understanding the influence of human activities on endangered species can be key to improving conservation efforts as well as the implementation of national strategies for sustainable development. Although the red panda is facing serious anthropogenic pressure (Acharya et al., 2018; Glatston et al., 2015; Panthi et al., 2017; Sharma, Belant, et al., 2014), previous studies did not thoroughly consider anthropogenic factors when modeling the habitat of this species (Kandel et al., 2015; Thapa et al., 2018). Consequently, anthropogenic impacts on the red panda and its habitat remain unclear, and a comprehensive assessment of the suitable habitat for red pandas in Nepal is not available. Due to insufficient information on red panda habitat at large spatial scales, conservation partners such as the government of Nepal, World Wildlife Fund, National Trust for Nature Conservation, and Red Panda Network have been unable to prepare effective policies, plans, and strategies for red panda conservation in Nepal.

In this study, we aim to assess human impact on endangered species living in high‐altitude regions in Nepal by using the red panda as an example. Our specific objectives are to (a) quantify suitable habitat for red pandas across Nepal; (b) determine the role of anthropogenic factors to predict suitable habitat for red pandas. The information from this study will be useful for the government of Nepal and conservation partners to prepare and implement policies, plans, and strategies for immediate and long‐term conservation of red panda in Nepal.

## 2 MATERIALS AND METHODS

### 2.1 Study area

**Figure 1:**
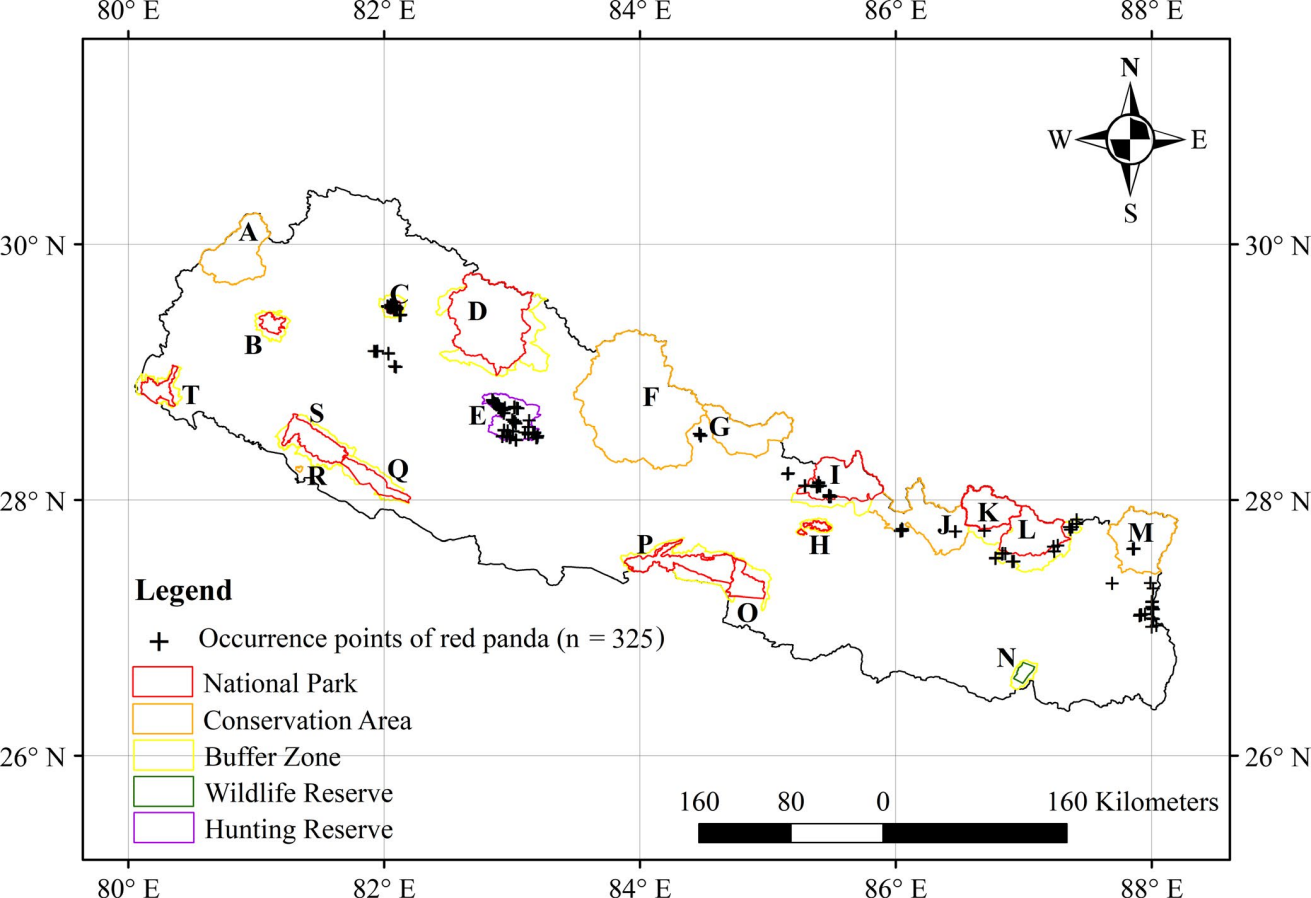
Distribution of protected areas in Nepal and the red panda occurrence points used to predict the suitable habitat in this study; A: Api Nampa Conservation Area, B: Khaptad National Park and its Buffer Zone, C: Rara National Park and its Buffer Zone; D: Shey Phoksundo National Park and its Buffer Zone; E: Dhorpatan Hunting Reserve; F: Annapurna Conservation Area; G: Manaslu Conservation Area; H: Shivapuri Nagarjun National Park and its Buffer Zone; I: Langtang National Park and its Buffer; J: Gaurishankar Conservation Area; K: Sagarmatha National Park and its Buffer Zone; L: Makalu Barun National Park and its Buffer Zone; M: Kanchenjunga Conservation Area; N: Koshi Tappu Wildlife Reserve and its Buffer Zone; O: Parsa National Park and its Buffer Zone; P: Chitwan National Park and its Buffer Zone; Q: Banke National Park and its Buffer Zone; R: Krishnasar Conservation Area, S: Bardia National Park and its Buffer Zone; T: Shuklaphanta National Park and its Buffer Zone (source of shape file of protected areas: UNEP‐WCMC & IUCN, 2017) and boundary of Nepal (Bjørn, 2009)

Nepal is situated in the central part of the Himalaya and covers an area of 147,181 km^2^. Nepal has diverse climates due to the large variation in elevation, varying from tropical lowlands in the south to alpine cold semi‐desert in the trans‐Himalayan zone (Ohsawa, Shakya, & Numata, 1986). The average annual rainfall is around 1,000–2,000 mm, but sometimes it exceeds 3,000 mm in some lower parts of the country (Ichiyanagi, Yamanaka, Murajic, & Vaidyad, 2007). Nepal has diverse geography ranging from very rugged and permanently snow and ice‐covered Himalayan Mountains in the north to tropical alluvial plains in the south. Due to variation in climate and topography, Nepal is classified into five physiographic zones (i.e., Terai, Siwalik, middle Mountain, high Mountain, and Himalaya; Barnekow Lillesø, Shrestha, Dhakal, Nayaju, & Shrestha, 2005; Shrestha, Shrestha, Chaudhary, & Chaudhary, 2010). In spite of economic obstacles, the government of Nepal has established 20 protected areas that cover more than 23% of the total land area of the country: 12 national parks, six conservation areas, one wildlife reserve, and one hunting reserve (Figure 1) (DNPWC, 2017). These protected areas provide natural habitat for elephant, musk deer, red panda, rhino, snow leopard, tiger, wild buffalo, wild dog, and other threatened wildlife (DNPWC, 2017).

### 2.2 Red panda occurrence data

We compiled two datasets including 30 first‐hand and 295 second‐hand red panda occurrence records (Figure 1). The second‐hand occurrence records were obtained from published research articles and unpublished government reports of Nepal. All second‐hand data were collected between 2009 and 2016 using a Global Positioning System (GPS). The sources of these second‐hand data are listed in Appendix 1. Based on the spatial distribution of the second‐hand data, we interviewed a number of red panda experts and local park rangers to identify other potential red panda habitats for primary data collection. We carried out fieldwork in September and October 2017 in Langtang National Park, Ilam, Panchthar, and Dhading districts of Nepal. In the field, the direct and indirect signs of red pandas (i.e., droppings) were recorded using a GPS by adopting the purposive sampling.

### 2.3 Environmental variables

#### 2.3.1 Bio‐climatic variables

Bio‐climatic variables were downloaded from the WorldClim database (http://worldclim.org/). The WorldClim database (version 2) is a set of 19 global bio‐climatic variables derived from over 4,000 weather stations between 1950 and 2000 with a spatial resolution of 1 km. The variables include annual time series with annual means, seasonality, and extreme or limiting temperature and precipitation data (Hijmans, Cameron, Parra, Jones, & Jarvis, 2005).

#### 2.3.2 Topographical variables

A digital elevation model (DEM) with a spatial resolution of 1 km was downloaded from the USGS website (https://earthexplorer.usgs.gov/; USGS/EarthExplorer, 2017), and the slope and aspect were derived from the DEM using ArcGIS software (ESRI, 2017).

#### 2.3.3 Vegetation‐related variables

Satellite‐derived normalized difference vegetation index (NDVI) is a commonly used vegetation index for ecological research. In this study, we used the NDVI time series to model red panda habitat. Since most of the secondary red panda occurrence data were collected between 2009 and 2013, we downloaded atmospherically corrected 10‐day composite NDVI images with a spatial resolution of 1 km over the same period (180 images, three images per month) acquired by SPOT4 and SPOT5 Vegetation (VGT) sensor from the European Space Agency product distribution portal (http://www.vito-eodata.be; Vito, 2017). We smoothed these NDVI images using an adaptive Savitzky–Golay filter in TIMESAT (Jönsson & Eklundh, 2004). The seasonal characteristics of five full phonological cycles were constructed based on the five years' time series NDVI data and statistical products (i.e., maximum, mean, minimum, standard deviation, and amplitude). The resulting smoothed data were used as environmental variables in our model. The forest cover data for the region were obtained from Advance Land Observing Satellite (http://www.eorc.jaxa.jp/ALOS/en; JAXA EORC, 2017). In addition, forest canopy height data with a 1‐km spatial resolution was obtained from the Spatial Data Access Tool (see https://webmap.ornl.gov/ogc/dataset.jsp?ds_xml:id=10023; Simard, Pinto, Fisher, & Baccini, 2011).

### 2.4 Anthropogenic variables

#### 2.4.1 Human population density

Human population density with a spatial resolution of 1 km was downloaded from the socio‐economic data and application center (http://sedac.ciesin.columbia.edu; CIESIN, 2000).

#### 2.4.2 Livestock density

Livestock (cattle, goat, and sheep) density with a spatial resolution of 1 km was obtained from the Center for Earth Observation and Citizen Science (see http://www.geo-wiki.org)” (Robinson et al., 2014).

#### 2.4.3 Distance to roads

Road networks were downloaded from the Geofabrik website (http://download.geofabrik.de/asia/nepal.html; OpenStreetMap Contributors, 2017). We then generated a raster file of the distance to roads with a spatial resolution of 1 km using ArcGIS (ESRI, 2017).

#### 2.4.4 Distance to paths

Path (tracks used by people and animals) networks were downloaded from the Geofabrik website (http://download.geofabrik.de/asia/nepal.html; OpenStreetMap Contributors, 2017). We then generated a raster file of the distance to paths with a spatial resolution of 1 km using ArcGIS (ESRI, 2017).

#### 2.4.5 Distance to human settlements

Settlement points throughout Nepal were obtained from the Department of Survey, Nepal. A raster layer of distance to human settlements with a spatial resolution of 1 km was created using ArcGIS (ESRI, 2017).

#### 2.4.6 Land cover and land use

Land use and land cover with a 1‐km spatial resolution were obtained from the Fine Resolution Observation and Monitoring Global Land Cover website (FROM‐GLC) (http://data.ess.tsinghua.edu.cn; Li et al., 2016).

### 2.5 Multicollinearity analysis

**Figure 2:**
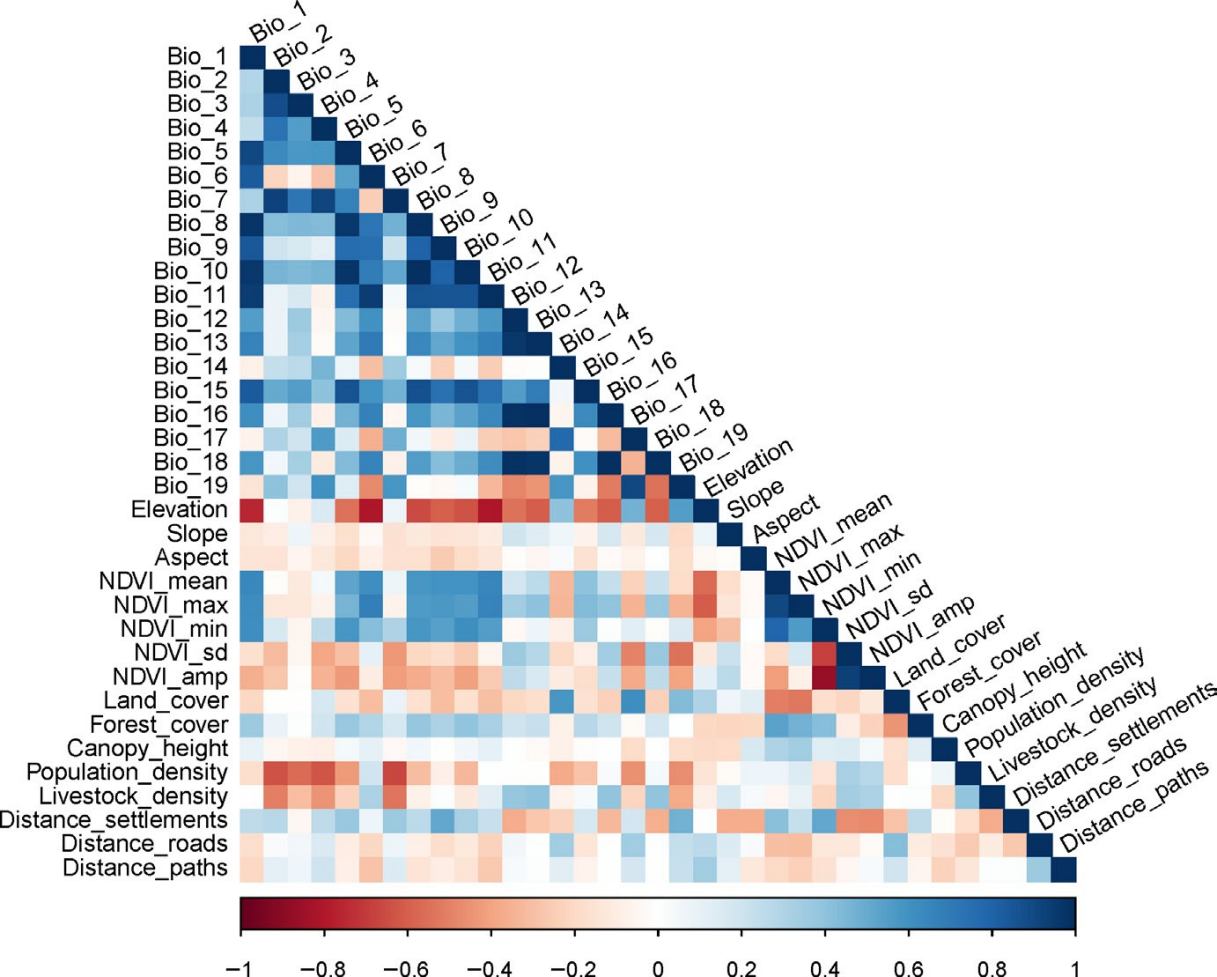
Correlation matrix of environmental and anthropogenic variables. Cool colored (blue) squares indicate a positive correlation and warm colored (red) squares indicate a negative correlation; darker colored squares indicate stronger correlation and paler colored squares indicate a weaker correlation

Removing the highly correlated (|*r*| > .70) variables for species distribution models is recommended for reliable and unbiased output (Braunisch et al., 2013; Dormann et al., 2013). We used ArcGIS to extract the values of these variables at species presence points (ESRI, 2017) and conducted a multicollinearity analysis between these variables using the ‘mctest’ package in R (R Core Team, 2018) (Figure 2). Finally, 18 highly correlated variables were removed from the dataset, and the remaining 17 variables were used for habitat modeling (Table 1).

**Table 1:** Environmental and anthropogenic variables used for modeling the red panda habitat suitability

Category	Data source	Variables	Abbreviation
Environmental	WorldClim	Annual mean temperature	Bio1
	WorldClim	Mean diurnal range	Bio2
	WorldClim	Temperature seasonality	Bio4
	WorldClim	Annual precipitation	Bio12
	WorldClim	Precipitation of driest month	Bio14
	USGS GTOPO30	Aspect	Aspect
	USGS GTOPO30	Slope	Slope
	SPOT‐VGT	Annual minimum NDVI	NDVI_min
	SPOT‐VGT	Standard deviation NDVI	NDVI_sd
	ALOS Japan	Forest cover	Forest_cover
	NASA EARTHDATA	Forest canopy height	Canopy_height
Anthropogenic	FROM‐GLC	Land use land cover	Land_cover
	NASA SEDAC	Human population density	Population_density
	Geofabrik	Distance to roads	Distance_roads
	Geofabrik	Distance to paths	Distance_paths
	Survey department Nepal	Distance to settlements	Distance_settlements
	Livestock Geo‐Wiki	Livestock density	Livestock_density

### 2.6 Ecological niche model

The maximum entropy (MaxEnt) model is one of the most reliable and robust model for species distribution and habitat suitability modeling (Phillips, Anderson, & Schapire, 2006). In addition, built‐in jackknife tests in the program allow users to estimate the significance of individual variables in computing the habitat suitability (Elith et al., 2006). We used the MaxEnt program version 3.4.0 (https://github.com/mrmaxent/Maxent) to develop environmental niche models. In this study, no primary and secondary data of red panda occurrence points were reported from two physiographical regions of Nepal: Terai and Siwalik. Therefore, these two regions were excluded from the current study to reduce modeling bias. The recommended default values were used for maximum iterations (1,000), while 10,000 background points were accepted (Barbet‐Massin, Jiguet, Albert, & Thuiller, 2012). We ran 10 replicates of each model.

### 2.7 Model scenarios, evaluation, and statistical analysis

We ran the model with two different scenarios to assess the impact of anthropogenic variables on red panda habitat prediction. First, we ran the model using only environmental variables. Next, we ran the model using both environmental and anthropogenic variables. Assessment of prediction accuracy is essential to validate the models and to understand model performance. We randomly selected fifty percent of the species occurrence points for training and used the other fifty percent to test both models. To evaluate the accuracy of the model predictions, we used both threshold‐independent and threshold‐dependent methods. For the threshold‐independent method, the area under the receiver‐operator curve (AUC) of models was reported (Phillips et al., 2006; Wiley, McNyset, Peterson, Robins, & Stewart, 2003). The higher the AUC, the higher the model performance was. An AUC < 0.7 indicates poor model performance, 0.7–0.9 indicates moderate performance, and >0.9 indicates excellent performance (Pearce & Ferrier, 2000). Although AUC is a commonly used model evaluation parameter, it is influenced by the geographic extent of the models (Lobo, Jiménez‐valverde, & Real, 2008). Therefore, we also used the threshold‐dependent method, that is, true skill statistic (TSS) to evaluate the accuracy of the model predictions (Allouche, Tsoar, & Kadmon, 2006; Merow, Smith, & Silander, 2013). True skill statistic was calculated for all model outputs (0–9 replications), and the final TSS was averaged from all 10 replicates. We tested the accuracy of the 10 replicates and found that they were normally distributed for all models (Shapiro–Wilk test, *p* = .05). Therefore, we used a *t *test (5% level of significance) to compare the differences in accuracy (i.e., AUC and TSS) between the model scenarios, as well as to ascertain the most accurate predictive model. Although the model accuracies may be affected by number of variables used to the model, we considered the best models those that had the highest accuracies.

The default logistic output of MaxEnt is a continuous variable ranging from 0 to 1, where high values indicate higher relative suitability. The maximum sum of the sensitivity and specificity (MaxSSS) threshold is appropriate to convert the continuous probability map to a binary map when only presence data are available (Liu, Newell, & White, 2016; Liu, White, & Newell, 2013). This is a widely used threshold that has been used in similar studies (Bista et al., 2018; Choe, Thorne, & Seo, 2016; KC et al., 2019). In this study, we used the MaxSSS threshold to generate the final suitable habitat maps.

## 3 RESULTS

### 3.1 Predicted suitable habitat with and without the use of anthropogenic variables

**Figure 3:**
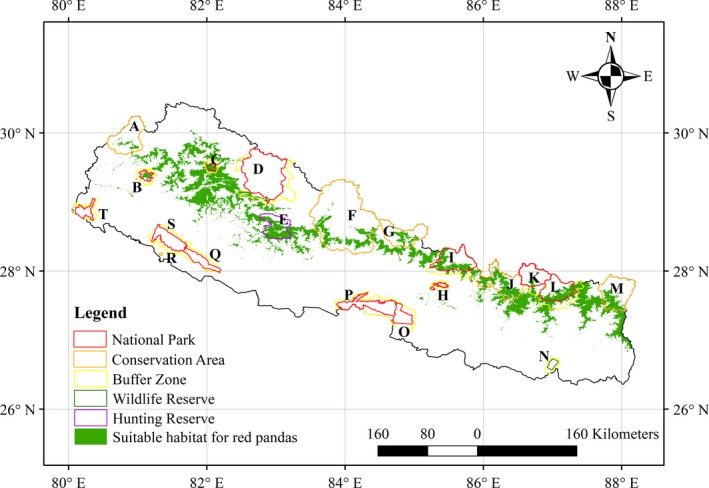
Predicted suitable habitat for red pandas based on the inputs of environmental variables only; A: Api Nampa Conservation Area, B: Khaptad National Park and its Buffer Zone, C: Rara National Park and its Buffer Zone; D: Shey Phoksundo National Park and its Buffer Zone; E: Dhorpatan Hunting Reserve; F: Annapurna Conservation Area; G: Manaslu Conservation Area; H: Shivapuri Nagarjun National Park and its Buffer Zone; I: Langtang National Park and its Buffer; J: Gaurishankar Conservation Area; K: Sagarmatha National Park and its Buffer Zone; L: Makalu Barun National Park and its Buffer Zone; M: Kanchenjunga Conservation Area; N: Koshi Tappu Wildlife Reserve and its Buffer Zone; O: Parsa National Park and its Buffer Zone; P: Chitwan National Park and its Buffer Zone; Q: Banke National Park and its Buffer Zone; R: Krishnasar Conservation Area, S: Bardia National Park and its Buffer Zone; T: Shuklaphanta National Park and its Buffer Zone (source of shape file of protected areas: UNEP‐WCMC & IUCN, 2017) and boundary of Nepal (Bjørn, 2009)

**Figure 4:**
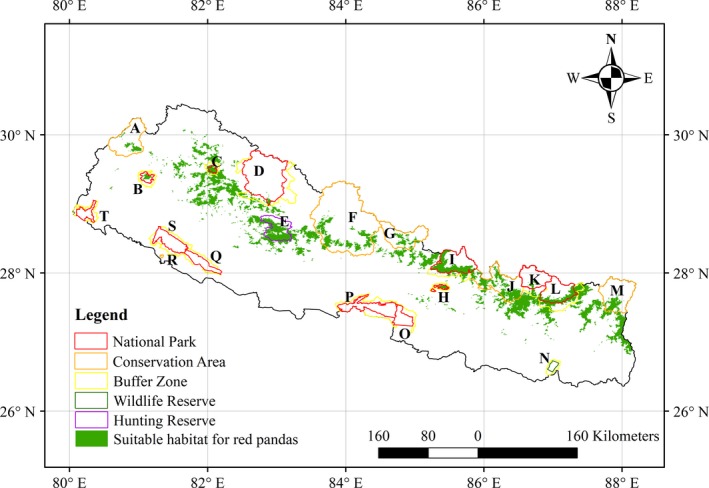
Predicted suitable habitat for red pandas based on the inputs of both environmental and anthropogenic variables; A: Api Nampa Conservation Area, B: Khaptad National Park and its Buffer Zone, C: Rara National Park and its Buffer Zone; D: Shey Phoksundo National Park and its Buffer Zone; E: Dhorpatan Hunting Reserve; F: Annapurna Conservation Area; G: Manaslu Conservation Area; H: Shivapuri Nagarjun National Park and its Buffer Zone; I: Langtang National Park and its Buffer; J: Gaurishankar Conservation Area; K: Sagarmatha National Park and its Buffer Zone; L:Makalu Barun National Park and its Buffer Zone; M: Kanchenjunga Conservation Area; N: Koshi Tappu Wildlife Reserve and its Buffer Zone; O: Parsa National Park and its Buffer Zone; P: Chitwan National Park and its Buffer Zone; Q: Banke National Park and its Buffer Zone; R: Krishnasar Conservation Area, S: Bardia National Park and its Buffer Zone; T: Shuklaphanta National Park and its Buffer Zone (source of shape file of protected areas: UNEP‐WCMC & IUCN, 2017) and boundary of Nepal (Bjørn, 2009)

The model based on the environmental variables identified a total of 18,193 km^2^ of suitable habitat for red pandas in Nepal (Figure 3). The model based on both environmental and anthropogenic variables identified a total of 13,781 km^2^ of suitable habitat for red pandas throughout Nepal (Figure 4). The performance of both models was robust, with high values for AUC (all > 0.93), as well as TSS (all > 0.74). However, the performance of the two models was significantly different (*p* < .05; *T *test). The model based on both environmental and anthropogenic variables performed better, with a relatively higher average TSS (0.7676 vs. 0.7485) (Table 2). Although the spatial distribution patterns of the two suitable habitat maps looked similar, it is notable that the total area of suitable red panda habitats predicted by both environmental and anthropogenic variables was much smaller, and more fragmented than the suitable habitat map predicted by the environmental variables only. The model based on both environmental and anthropogenic variables showed that approximately 60% of the suitable red panda habitats were located outside the existing protected areas of Nepal. Out of the 13,781 km^2^ of red panda habitat, 5,578 km^2^ were located inside the existing 13 protected areas, with the remaining 8,203 km^2^ located outside the protected areas. The Langtang National Park covers the highest portion of suitable red panda habitat in comparison to other existing protected areas.

**Table 2:** Comparison of the model performance in predicting the suitable habitat for red pandas in Nepal

Model	AUC		TSS	
	Mean	*SD*	Mean	*SD*
Environmental variables	0.9300^a^	0.0067	0.7485^a^	0.0236
Environmental and anthropogenic variables	0.9454^b^	0.0103	0.7676^b^	0.0295

Note

For each model scenario, the AUC and TSS were given as the average values of ten replicates. Superscript letters indicate significant differences among the means of AUC and TSS. Different superscript letters indicate significant differences at *p* < .05 (*T *test).

### 3.2 Variables affecting red panda habitat suitability at a national level

**Figure 5:**
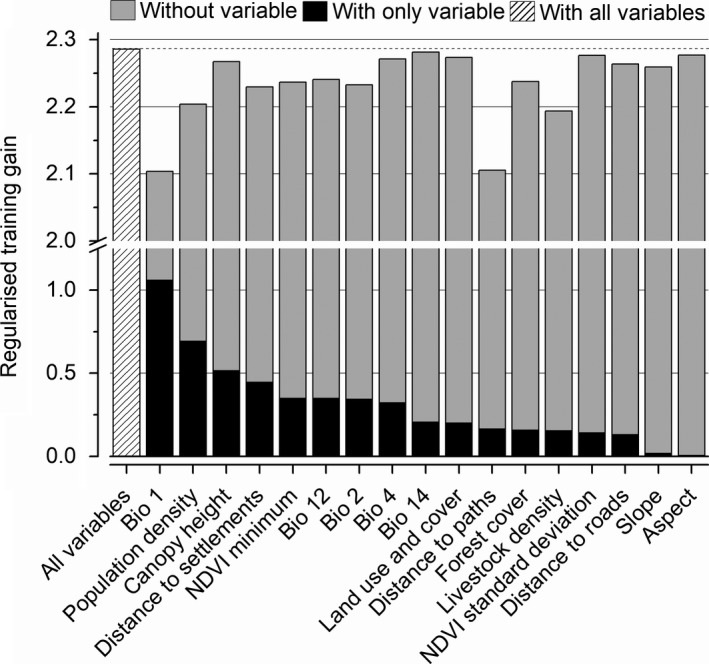
Importance of environmental and anthropogenic variables in modeling the current suitable habitat for red pandas in Nepal. The regularized training gain describes how much better the model distribution fits the presence data compared to a uniform distribution. “Without variable” indicates the effect of removing a specific single variable from the full model. “With only variable” indicates the results of the model when a single variable is run in isolation. “With all variables” indicates the results of the model when all variables are run

Analysis of the contribution of environmental and anthropogenic variables to the predictive model indicated that distance to paths, annual mean temperature (Bio1), livestock density, and human population density were the most important variables contributing to the prediction of suitable red panda habitat in Nepal (Figure 5). It is notable that among these top four variables, three of them are anthropogenic variables. We also found that variables such as the canopy height, land use and land cover, standard deviation of NDVI, distance to roads, slope, aspect, temperature seasonality (Bio4), and the precipitation of driest month (Bio14) barely contributed to the prediction of suitable habitat for red pandas at a large spatial scale in Nepal. The remaining five variables, including forest cover, distance to settlements, NDVI minimum, mean diurnal range (Bio2), and annual precipitation, had a moderate contribution to the model prediction.

**Figure 6:**
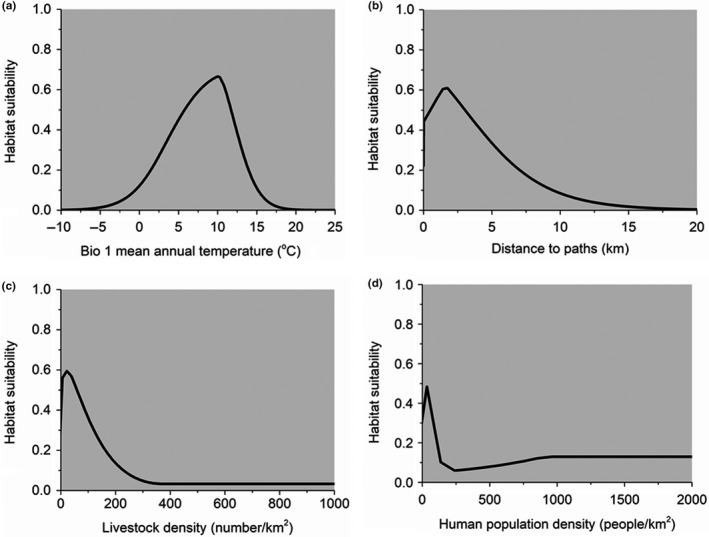
Response curves showing the relationship between habitat probability of red pandas and the top four contributed variables. The response curves were derived from the model using both environmental and anthropogenic variables

The response curves of the top four variables contributing to the prediction of red panda habitat (Figure 6) indicate that the optimal habitat for red pandas occurred in areas where the mean annual temperature (Bio1) was between 5°C and 10°C (Figure 6a). The probability of suitable habitat for red pandas increased with increasing distance to the nearest paths, but decreased dramatically after approximately 2 km from the paths (Figure 6b). The relationships between red panda habitat suitability and livestock density and human population density were negative (Figure 6c,d). An increase in livestock density, as well as human population density, significantly reduced habitat suitability for red pandas.

## 4 DISCUSSION

We successfully predicted suitable habitat for red pandas in Nepal using both environmental and anthropogenic variables. Our results show that three out of the four top predictor variables are anthropogenic factors, that is, the distance to paths, livestock density, and human population density, which all have a negative impact on red panda habitat suitability. Nepal is famous for tourism and several tourist routes, and paths for recreational trekking have been constructed in the high‐altitude regions near red panda habitat. In Nepal, many local people also live in very high mountains (Chidi, 2009). These people manage the facilities for tourists and use these paths for their daily livelihood such as fuelwood and forest products collection. If the flow of local people and tourists increase, the negative impact of human paths may increase significantly in the near future. In addition to tourism, livestock is an important source of cash income for farm households in the high mountains of Nepal. However, a number of local‐level studies have reported that livestock grazing has a negative impact on red pandas (Acharya et al., 2018; Sharma, Belant, et al., 2014; Yonzon & Hunter, 1991b). This is part of a larger trend of livestock grazing contributing to biodiversity loss around the world (Alkemade et al., 2013). For example, in China, free‐ranging livestock consumes considerable amounts of bamboo, which is partly responsible for the degradation of the giant panda habitat (Hull et al., 2014; Li, Pimm, Li, Zhao, & Luo, 2017). Livestock grazing also has a negative impact on grouse populations worldwide (Dettenmaier, Messmer, Hovick, & Dahlgren, 2017). Similarly, our study revealed that the high livestock density has a significant negative impact on red panda habitat at a large spatial scale in Nepal.

Biodiversity is facing serious anthropogenic impacts and is declining rapidly throughout the world (Maxwell et al., 2016; Tittensor et al., 2014). There is growing evidence that human population growth is a major cause of wildlife loss (WWF, 2018). Therefore, it is not surprising that we identified human population density as one of the top predictor variables contributing to the prediction of suitable habitat for red pandas across Nepal. This presents a significant conservation challenge; on the one hand, the people living in the high‐altitude regions of Nepal depend on livestock and tourism for their livelihoods. On the other hand, human activities are threatening the red panda and its habitat. We recommend that the Department of National Parks and Wildlife Conservation should coordinate with the Department of Livestock Services and Department of Tourism to mitigate the impacts of livestock and tourist routes on red panda. We also recommend promulgating legislation to allow livestock in meadows but not the forest with understory bamboo, and to prohibit the collection of fodder and fuelwood from core habitat of red panda to manage the local people and wildlife in a win‐win situation.

In our study, we used both environmental and anthropogenic variables to achieve a more accurate and reliable prediction of suitable habitat for red panda. We estimated that approximately 13,800 km^2^ of suitable habitats are available for red pandas in Nepal, which is significantly lower than the previous studies conducted by Kandel et al. (2015) and Thapa et al. (2018), who reported 17,400 km^2^ and 20,150 km^2^ of suitable habitat for red pandas, respectively. These studies only used bio‐climatic and topographical variables to model suitable habitat and failed to consider anthropogenic and vegetation‐related variables. Red panda presence has been previously confirmed in only seven of the protected areas of Nepal: Kanchenjunga Conservation Area (Kandel et al., 2015), Makalu Barun National Park (Bista et al., 2018; MBNP, 2016), Sagarmatha National Park (Mahato, 2004), Gaurishankar Conservation Area (Thapa, 2016), Langtang National Park (Thapa & Basnet, 2015; Yonzon & Hunter, 1991a, 1991b), Dhorpatan Hunting Reserve (Panthi et al., 2012, 2015, 2017), and Rara National Park, Nepal (Sharma, Belant, et al., 2014; Sharma, Swenson, et al., 2014). In this study, we predicted that there is suitable red panda habitat has inside 13 protected areas of Nepal, but we found that only 40% of predicted suitable habitat is covered by the existing protected areas. The suitable red panda habitat patches between Kanchenjunga Conservation Area and Makalu Barun National Park, Langtang National Park and Manaslu Conservation Area, Annapurna Conservation Area and Dhorpatan Hunting Reserve, and habitats around the Rara National Park are still unprotected. The Department of Forests and Soil Conservation of Nepal is responsible for managing and protecting wildlife and their habitats outside protected areas. However, the major focus of this department has been on timber production and watershed management. The Department of Forests and Soil Conservation cannot conserve wildlife as effectively as protected areas with existing resources. Therefore, enhancing the department's capacity to protect the red panda and other wildlife, as well as protecting habitat outside current protected areas should be high priorities. Although the presence of red panda was scientifically confirmed from most parts of the suitable habitat identified by this study, they have not been documented or confirmed by the Khaptad National Park, Shivapuri Nagarjun National Park, Api Nampa Conservation Area, and Manaslu Conservation Area. These protected areas could be a suitable destination for red panda translocations to reduce the risk of red panda extinction. For instance, our study identified 55 km^2^ of suitable red panda habitat in Shivapuri Nagarjun National Park. As this park is the closest protected area to Kathmandu, the capital city of Nepal, this could also help attract wildlife tourists.

This study identified suitable habitat for red panda in patches of varying size. In addition to conserving large habitat patches, restoring the unsuitable area around small habitat patches and improving habitat quality is recommend for long‐term conservation of the red panda. Similar to the recommendation of Bista et al. (2019), we recommend preparing and implementing site‐specific conservation plans to conserve this species and its habitat. Although this study only considered a single species, we showed that wildlife of the Himalayan region faces anthropogenic pressure. Conservationists should pay more attention to this region for the conservation of specific species and overall biodiversity. In the future, researchers should also identify the impacts of other factors like climate and land use change on red pandas.

The modeling was done with presence only data, so this study couldnot account the imperfect detection of the species. We are not modeling the probability of occurrence of red pandas but rather an index of their habitat suitability, due to the lack of absence data. We used only one sample (presence point of red panda) from one grid having one‐km resolution to lessen spatial autocorrelation.

## CONFLICT OF INTEREST

None declared.

## AUTHOR CONTRIBUTIONS

S.P. and T.W. conceived the project and designed the study. S.P., T.W., and A.T. collected the occurrence points. S.P., T.W., and Y.S. analyzed data interpreted the results. S.P. and T.W. wrote the manuscript. All authors critically reviewed the manuscript.

### OPEN DATA BADGES

This article has earned an https://openscience.com for making publicly available the digitally‐shareable data necessary to reproduce the reported results. The data is available at https://figshare.com/articles/Occurrence_points_of_red_panda_xlsx/9962552.

## Data Availability

https://figshare.com/articles/Occurrence_points_of_red_panda_xlsx/9962552

## APPENDIX 1

### Sources of secondary red panda occurrence data

**Table:**

Location	Number of red panda presence points	Source
Nepal	22	Kandel et al. (2015)
Rara National Park	73	Sharma (2013)
Jumla district	9	Bhatta, Shah, Devkota, Paudel, & Panthi (2014)
Dhorpatan Hunting Reserve	117	Panthi (2011)
Manang district	5	Paudel (2009)
Langtang National Park	14	Chalise (2013)
Langtang National Park	30	Kathayat (2016)
Gaurishankar Conservation Area	9	Thapa (2016)
Makalu Barun National Park	15	MBNP (2016)
Ilam district	1	Chalise (2009)
Total	295	
